# Caldendrin represses neurite regeneration and growth in dorsal root ganglion neurons

**DOI:** 10.1038/s41598-023-29622-9

**Published:** 2023-02-14

**Authors:** Josue A. Lopez, Annamarie Yamamoto, Joseph T. Vecchi, Jussara Hagen, Kyungmoo Lee, Milan Sonka, Marlan R. Hansen, Amy Lee

**Affiliations:** 1 Department of Neuroscience, University of Texas-Austin, 100 E. 24th St., Austin, TX 78712 USA; 2 Department of Molecular Physiology and Biophysics and Otolaryngology Head-Neck Surgery, Iowa Neuroscience Institute, Pappajohn Biomedical Institute, University of Iowa, Iowa City, Iowa 52242 USA; 3 Electrical and Computer Engineering, Iowa Institute for Biomedical Imaging, University of Iowa, 51 Newton Rd. Iowa City, Iowa, 52242 USA

**Keywords:** Cell biology, Neuroscience, Physiology

## Abstract

Caldendrin is a Ca^2+^ binding protein that interacts with multiple effectors, such as the Ca_v_1 L-type Ca^2+^ channel, which play a prominent role in regulating the outgrowth of dendrites and axons (*i.e*., neurites) during development and in response to injury. Here, we investigated the role of caldendrin in Ca_v_1-dependent pathways that impinge upon neurite growth in dorsal root ganglion neurons (DRGNs). By immunofluorescence, caldendrin was localized in medium- and large- diameter DRGNs. Compared to DRGNs cultured from WT mice, DRGNs of caldendrin knockout (KO) mice exhibited enhanced neurite regeneration and outgrowth. Strong depolarization, which normally represses neurite growth through activation of Ca_v_1 channels, had no effect on neurite growth in DRGN cultures from female caldendrin KO mice. Remarkably, DRGNs from caldendrin KO males were no different from those of WT males in terms of depolarization-dependent neurite growth repression. We conclude that caldendrin opposes neurite regeneration and growth, and this involves coupling of Ca_v_1 channels to growth-inhibitory pathways in DRGNs of females but not males.

Unlike most neurons in the central nervous system (CNS), neurons of the peripheral nervous system (PNS) can regenerate their neurites following injury^1,2^. The transition to a growth-competent state is thought to involve the re-activation of developmental pathways, which enables injured nerves to overcome a variety of factors that normally oppose neurite outgrowth. Ca^2+^ ions are critical second-messengers in this process, and can have positive or negative effects on neurite regeneration, growth, and pathfinding^3,4^. The downstream mechanisms whereby Ca^2+^ ions regulate the neurite growth dynamics are poorly understood.

One model posits that a decline in electrical activity following the separation of peripheral nerves from their targets causes a reduction in Ca^2+^ influx through voltage-gated Ca^2+^ channels, which is needed to support neurite regeneration^5–7^. Multiple lines of evidence implicate Ca_v_1 L-type channels in this context. Ca_v_1 antagonists facilitate the regrowth of axons in vitro^5,8^ and genetic disruption of the Ca_v_1.2 channel promotes recovery from peripheral nerve injury in vivo^5^. A reduction in Ca_v_1-mediated Ca^2+^ signals could repress transcription of growth-inhibiting genes^5^ and/or facilitate membrane trafficking and cytoskeletal assembly within the growth cone of the regenerating neurite^9,10^.

Ca_v_1 channels interact with a variety of proteins that could transduce the inhibitory effects of Ca_v_1-mediated Ca^2+^ signals on neurite growth. An important candidate in this regard is caldendrin—a member of a family of Ca^2+^ binding proteins (CaBPs) that are highly expressed in the retina and inner ear^11–14^. In the central nervous system, caldendrin is highly expressed in subsets of neurons^13,15^ and plays an important role in synaptic plasticity and fear conditioning^16,17^. Caldendrin and its shorter splice variants, CaBP1-S and CaBP1-L, bind to a site in the C-terminal domain of Ca_v_1.2 and Ca_v_1.3 channels that also binds calmodulin (CaM)^14,18–20^. This interaction with caldendrin potentiates the opening of Ca_v_1 channels^14^. In cochlear spiral ganglions of caldendrin knockout (KO) mice, enhanced regenerative growth of neurites correlates with a reduction in Ca_v_1 Ca^2+^ signals^21^.

Here, we tested the hypothesis that caldendrin is a fundamental regulator of Ca_v_1-dependent mechanisms constrain neurite regeneration in the PNS. To test this hypothesis, we analyzed the expression of caldendrin in dorsal root ganglion neurons (DRGNs) and compared neurite growth in cultures of DRGNs from wild-type (WT) and caldendrin knockout (KO) mice.

## Results

### Caldendrin is expressed in medium and large diameter DRGNs

**Figure 1:**
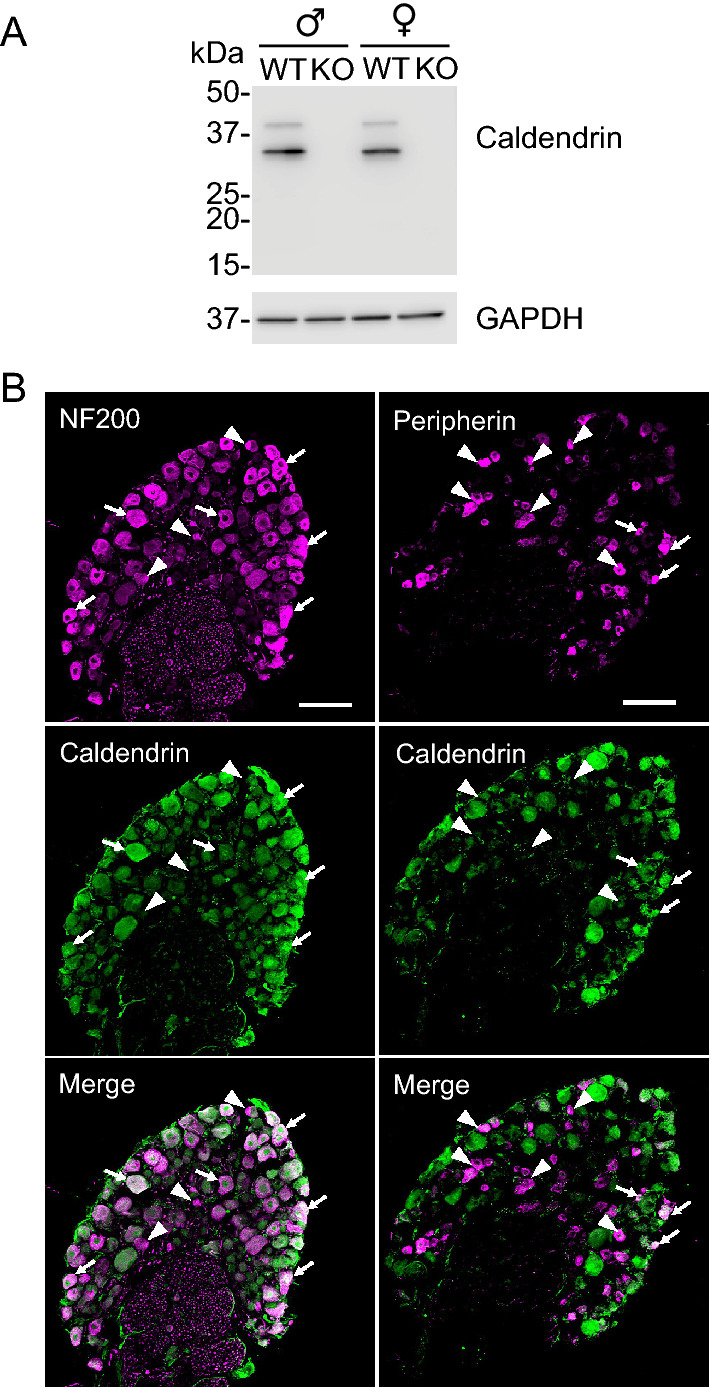
Caldendrin is expressed primarily in myelinated DRGNs. (**A**) Western blots of DRG lysates from WT and caldendrin KO mice probed with antibodies that recognize all CaBP1 variants (upper panel) or GAPDH to ensure equal loading of protein between lanes (lower panel). 36- and 33- kDa bands corresponding to caldendrin were detected in WT but not in caldendrin KO lysates whereas bands corresponding to CaBP1 variants were not observed. The gel images correspond to a single blot that was sequentially probed with anti-caldendrin and anti- GAPDH antibodies (Supp. Figure S1A). (**B**) Confocal micrographs of DRG cryosections from WT mouse double-labeled with antibodies against caldendrin and NF200 or peripherin. Arrows and arrowheads indicate cells in which caldendrin is or is not, respectively, co-localized with NF200 or peripherin. Results are representative of at least 3 independent experiments. Scale bar, 100 µm.

Alternative splicing gives rise to multiple CaBP1 variants of which caldendrin is the major variant expressed in the CNS^13,15^ (Fig. 1A). By Western blot, caldendrin can be detected as 38 kDa and 33 kDa forms, which may arise from alternate translational start sites^13,15^. Using antibodies that specifically recognize caldendrin (*i.e.,* do not produce immunoreactivity in caldendrin KO mice^15^), we detected both ~ 38 kDa and 33 kDa proteins in mouse DRG lysates. Of the two caldendrin variants, the 33-kDa band was more intense in DRG lysates obtained from males or females. The specificity of the immunolabeling was illustrated by the absence of both bands in DRG lysates from the caldendrin KO mice (Fig. 1A, Supplementary Fig. S1A).

DRGNs are a heterogeneous population of neurons that mediate various somatosensory modalities including the sense of touch, temperature, pain, and body position. To gain insights into which subpopulations of DRGNs express caldendrin, we double-labeled DRG tissue sections with antibodies against caldendrin and neurofilament 200 heavy chain (NF200) or peripherin which are expressed in medium to large non-nociceptive neurons (*i.e.,* myelinated, A-β fibers^22^) and small nociceptive neurons (*i.e*., C-fiber and A-δ fibers^23^), respectively. Caldendrin immunolabeling was diffusely localized in the soma and occasionally in nuclei of medium and large DRGNs. Nuclear localization of caldendrin has been reported previously in neurons^15,19^, and may be related to the roles of caldendrin in regulating transcription^24^. The vast majority of DRGNs that were labeled with caldendrin antibodies were NF200-positive (~ 92% of 224 cells); only ~ 5% (of 186 cells) were peripherin-positive (n = 3 independent experiments) (Fig. 1B). These results show that caldendrin is expressed in DRGNs and is localized primarily in myelinated A-β fibers.

### Regenerative neurite growth is enhanced in DRGNs from caldendrin KO mice

**Figure 2:**
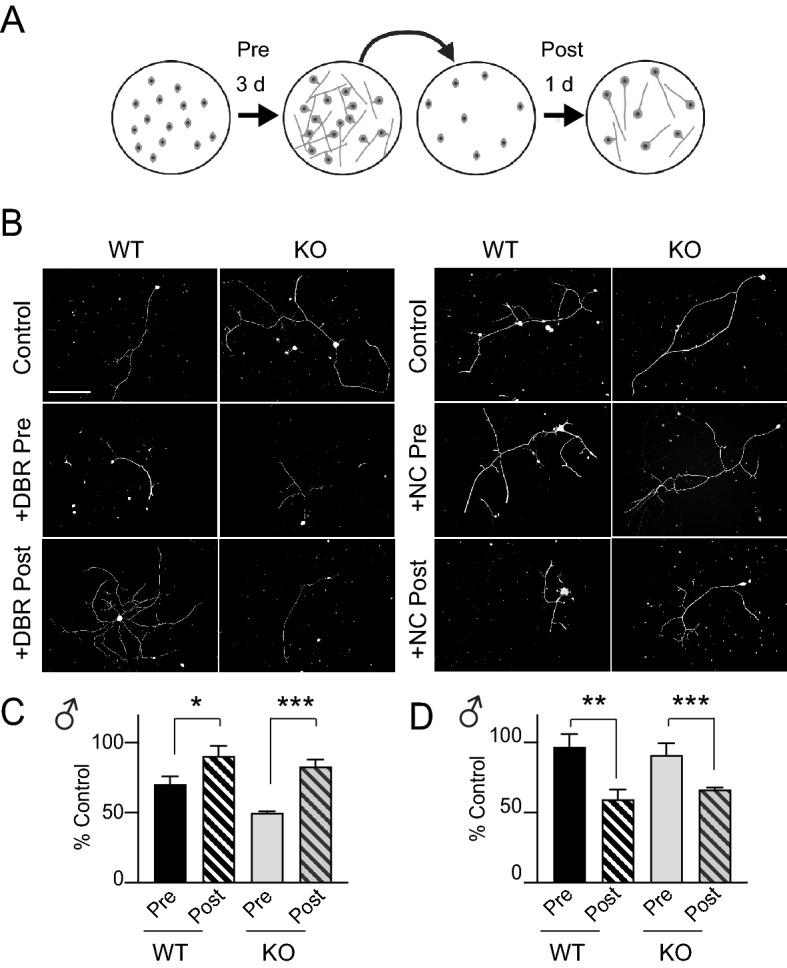
In vitro axotomy assay for analyses of neurite regeneration and growth. (**A**) Schematic of the in vitro axotomy assay. DRGNs were cultured for 3 d prior to replating and culture for 1 d before immunolabeling with NF200 antibodies. (**B**) Representative confocal images of DRGNs from WT or caldendrin KO male mice cultured with control medium or medium containing DBR (40 µM) or nocodazole (NC, 50 µM) before (Pre) or after (Post) replating. Scale bars, 500 µm. (C,D) Maximal neurite length per DRGN in cultures exposed to DBR (**C**) or NC (**D**) was normalized to that in the control group (no drug) and plotted as % Control. Each point represents the average obtained from n = 33 DRGNs from 3 independent cultures. Error bars represent SEM. In C, *p = 0.0187, t(4) = 3.83; ***p = 0.0004, t(4) = 11.02. In D, **p = 0.005, t(4) = 5.602; ***p = 0.0073, t(4) = 5.032. Significant differences were determined by unpaired t-tests.

Within 24 h of dissociation and culture, DRGNs from adult rats develop compact arborizing neurites prior to an elongating, less branched phase of neurite growth by 48 h^25^. These distinct phases can be simulated in an in vitro axotomy assay in which DRGNs from naive mice are cultured for 3 days prior to replating. The initial 3 days following dissociation mimics a transcription-dependent, conditioning response to nerve injury that is suppressed by the RNA polymerase II inhibitor, 5,6-dichlorobenzimidazole riboside (DBR). After replating, the DRGN neurites exhibit an elongating pattern of growth that is blunted by the microtubule destabilizer nocodazole^26^ (Fig. 2A). Consistent with the previous characterization of this assay^26^, DRGN neurite growth in WT and caldendrin KO cultures was strongly inhibited by the administration of DBR before, and nocodazole after, the replating step (Fig. 2B–D).

**Figure 3:**
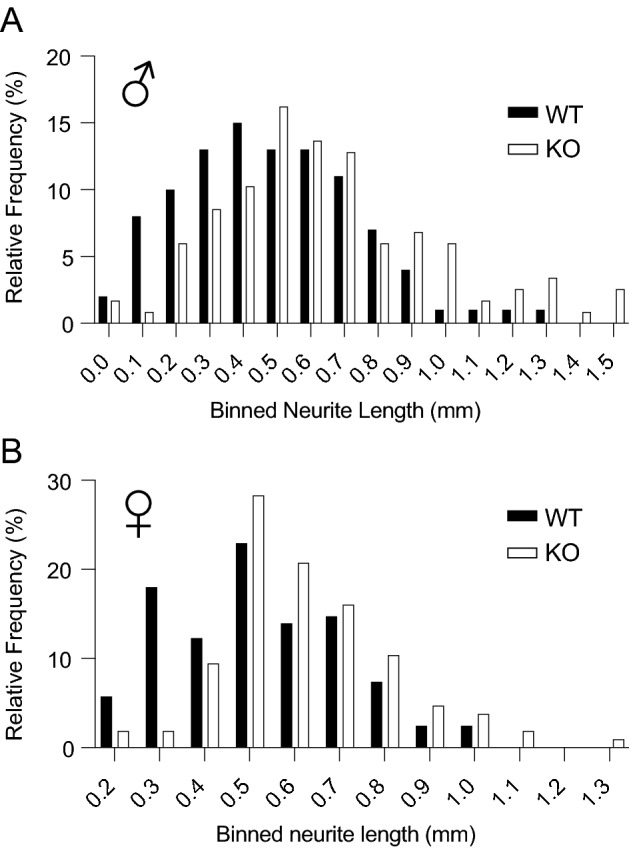
Caldendrin represses neurite regrowth after in vitro axotomy. DRGNs from WT and caldendrin KO mice were subject to in vitro axotomy and neurite lengths measured 1 d after replating as in Fig. 2. (**A**,**B**) Frequency distributions of maximal neurite lengths in cultures from male (**A**) and female (**B**) mice. Binned neurite length values represent the center of the bin. Data were collected from images of individual DRGNs (n = at least 32 images per culture, in 3 independent cultures). There was a significant difference in distributions for WT and caldendrin KO mice (*p* = 0.002 for males, *p *= 0.001 for females by Kolmogorov–Smirnov test, n = 3 mice in each group).

Having validated the in vitro axotomy assay for our studies, we next tested whether caldendrin impinges on neurite regeneration and outgrowth in DRGNs as in spiral ganglion neurons^21^. If so, then DRGNs from caldendrin KO mice should regrow their neurites after replating more robustly than those from WT mice. To test this prediction, we compared the distribution of neurite lengths of WT and caldendrin KO DRGNs 1 d after replating. Indeed, there was a significant rightward shift in the frequency distribution of neurite lengths in cultures from caldendrin KO mice compared to WT mice (Fig. 3A,B).

**Figure 4:**
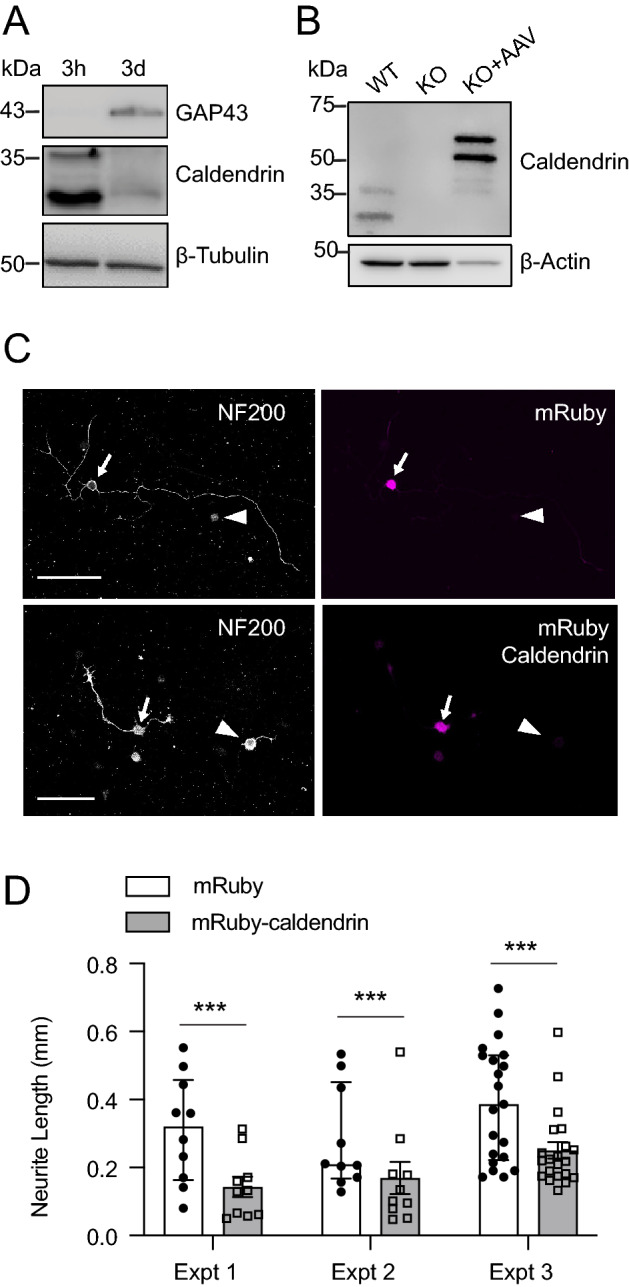
Reductions in caldendrin expression correlate with enhanced neurite growth in cultured DRGNs. DRGNs were plated in the in vitro axotomy assay for analysis of protein expression levels (**A**,**B**), or neurite growth (**C**,**D**). (**A**) Western blot showing decline in caldendrin protein levels when GAP43 expression is high. DRGN cultures from WT male mice were lysed 3 h or 3 d after plating and subject to electrophoresis. The western blot images correspond to 3 different gels in which lysates for the 2 time points were run in the same gel prior to probing with anti-caldendrin, anti- GAP43, or anti-β-Tubulin antibodies (Supp. Figure S1B). (**B**) Western blot of lysates from cultures exposed to AAV-mRuby (control) or AAV-mRuby-caldendrin for 3 d prior to harvest. Samples correspond to those from WT mice (lane 1) or caldendrin KO mice (lanes 2,3). Lane 3 represents caldendrin KO culture treated with AAV expressing mRuby-tagged caldendrin which is detected as two bands of higher molecular weight than the native caldendrin variants in WT lysates. Western blot images were cropped from a single blot that was probed sequentially with anti-caldendrin and anti-ß-actin antibodies. (**C**) Confocal micrographs of caldendrin KO cultures infected with AAV-mRuby (top) or AAV-mRuby-caldendrin (bottom) showing immunolabeling for NF200 (left) and mRuby (right). NF200-positive DRGNs with and without mRuby fluorescence are indicated by arrows and arrowheads, respectively. Scale bar, 100 µm. (**D**) Maximal neurite length per DRGN was measured in replated caldendrin KO cultures following AAV-mediated expression of mRuby (control) or mRuby-caldendrin. Data are shown for 3 independent experiments. Bars represent mean ± SEM. There was a significant effect of AAV treatment group on neurite length (F (1, 78) = 20.48, *p* < 0.0001, by 2-way ANOVA); ***p = 0.0003 by post-hoc Tukey’s test.

Peripheral nerve injury induces the expression of regeneration-associated genes (RAGs)^27^, which is thought to prime DRGNs for the elongating phase of neurite outgrowth^25^. One such RAG encodes growth associated protein 43 (GAP43, a.k.a. neuromodulin)—a CaM binding protein that regulates actin dynamics and facilitates neurite growth during development and regeneration^28^. In the replating assay, GAP43 and other RAGs are upregulated in the initial 3-day culture period^26^. By western blot, we noted a time-dependent increase in GAP43 expression and corresponding decrease in caldendrin expression between 3 h and 3 d in WT cultures (Fig. 4A, Supplementary Fig. S1B). If downregulation of caldendrin is necessary for induction of RAGs that promote neurite growth, then increasing caldendrin expression in caldendrin KO DRGNs during the transcription-dependent phase of the replating assay should suppress neurite growth. To test this, we used adeno-associated virus (AAV) to overexpress an mRuby-tagged caldendrin or mRuby control in caldendrin KO DRGNs during the 3 days prior to replating. With this approach, the mRuby-tagged caldendrin was detected as ~ 55- and 65- kDa proteins by western blot, which was consistent with the addition of the mRuby tag to the 33- and 36 kDa variants of caldendrin (Fig. 4B, Supplementary Fig. S1C). As predicted, neurite lengths were shorter in caldendrin KO cultures treated with AAV-mRuby-caldendrin than with the AAV-mRuby (Fig. 4C,D). These results support a cell autonomous mechanism whereby caldendrin represses neurite regrowth following in vitro axotomy.

**Figure 5:**
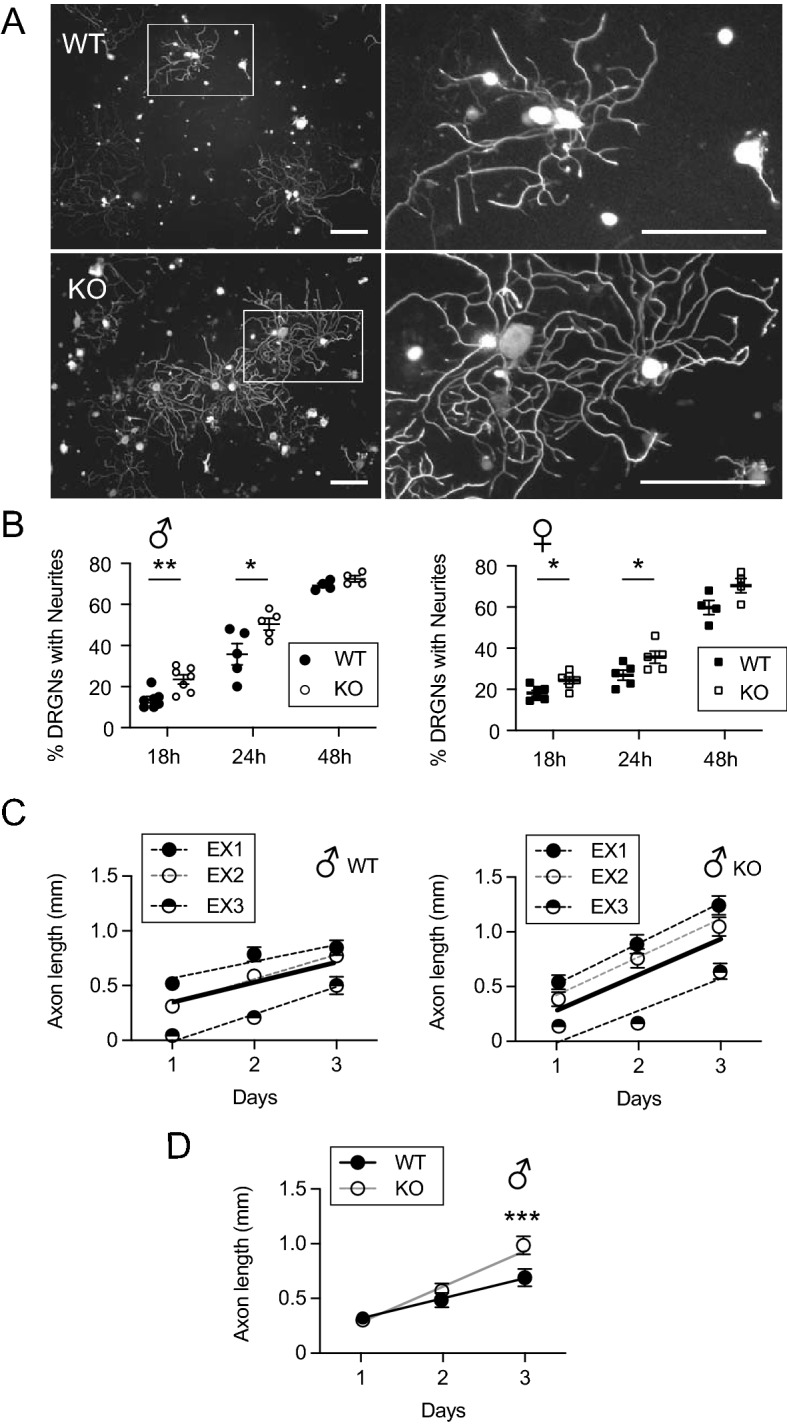
Caldendrin inhibits the regeneration and rate of outgrowth of DRGN neurites. DRGNs from WT and caldendrin KO mice were cultured for various periods prior to immunolabeling with NF200 antibodies. (**A**) Representative confocal images of WT and caldendrin KO DRGNs (24 h in culture). Boxed region in left panels is shown at higher magnification in right panels. Scale bars, 200 µm. (**B**) Percent of DRGNs with neurites in cultures from males (left) and females (right) at the indicated times in culture. Each point represents the average obtained from a single culture (n = 300–400 DRGNs/culture), bars represent mean ± SEM. For male data: **t(12) = 3.584, *p* = 0.004; *t(8) = 2.447, *p* = 0.04. For female data: *t(10) = 2.916, *p* = 0.015; *t(8) = 2.262, *p* = 0.05, compared to WT by unpaired t-test. (**C**,**D**) Neurite growth measured in DRG explants. DRGs were cultured from WT/PirtGCaMP3 (left) and caldendrin KO/PirtGCaMP3 (right) male mice (at least 2 DRGs per mouse, n = 3 mice, results from each mouse are represented in different symbols). Lengths of the same neurites were measured each day for 3 days. Dashed and smooth lines represent linear fits from individual explants and the averaged data, respectively. Symbols and bars represent mean ± SEM. Data from *C* are replotted in *D* to indicate significant increase in neurite length in caldendrin KO explants on day 3. ***p = 0.0006, determined by Kolmogorov–Smirnov Test.

To determine whether caldendrin affects the regeneration and/or the continued outgrowth of new neurites, we compared the time-dependence of neurite growth in WT and caldendrin KO DRGNs. First, we analyzed the number of DRGNs that had regrown neurites at various timepoints after dissociation. The percentage of DRGNs with neurites was significantly higher at 18 h and 24 h but not 48 h in cultures obtained from caldendrin KO vs WT mice (Fig. 5A,B). Next, we analyzed the rate of regrowth of individual neurites using DRG explants. We plated the explants on micropatterned substrates such that the growth of individual neurites within the grooves of the pattern could be monitored over time. In these experiments, the rate of neurite growth was 2 times faster in explants from caldendrin KO mice (0.35 ± 0.03 mm/day, n = at least 2 explants from 3 mice) than in explants from WT mice (0.17 ± 0.03 mm/day, n = at least 2 explants from 3 mice; *p* = 0.0002 by analysis of covariance) (Fig. 5C). Moreover, neurites were significantly longer in explants from caldendrin KO than from WT mice by day 3 in culture (Fig. 5D, Supplementary Fig. S2). These findings suggest that caldendrin acts as a brake on both the regeneration and outgrowth of neurites of DRGNs in dissociated and explant cultures.

### A role for caldendrin in the Ca_v_1-dependent repression of neurite growth

**Figure 6:**
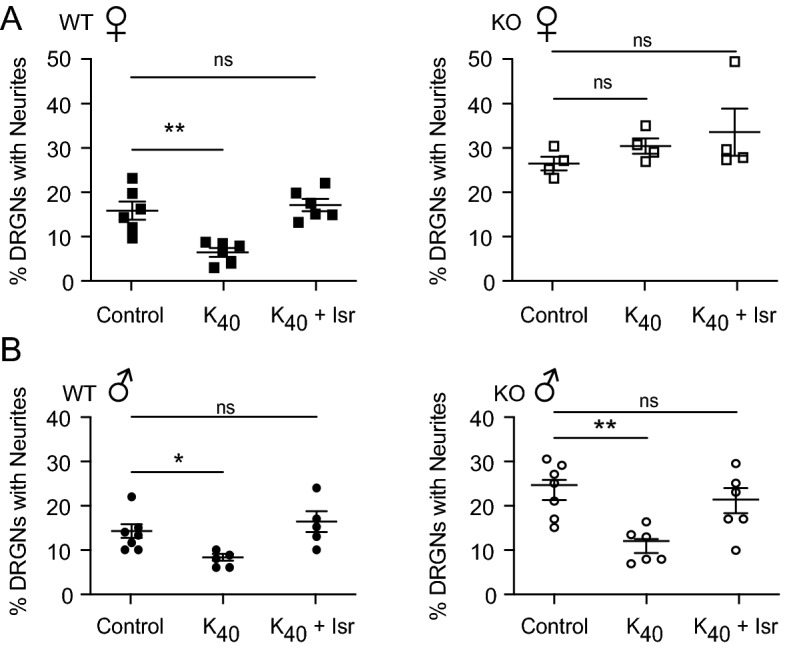
Caldendrin is required for Ca_v_1-dependent repression of neurite growth in DRGNs of females but not males. (**A**,**B**) DRGNs were cultured for 18 h with or without 40 mM KCl (K_40_) ± isradipine (Isr, 10 µM). Analysis of % DRGNs with neurites as in Fig. 5 in cultures from WT or caldendrin KO females (**A**) or males (**B**). Each point represents average determined from ~ 300 DRGNs per culture. Bars represent mean ± SEM. (**A**) F(2,15) = 14.69 ,**p = 0.001; F(2,9) = 1.13, *p* = 0.6;l (**B**) F(2,14) = 5.53 ,*p = 0.05; F(2,16) = 8.1 ,**p = 0.002, by one-way ANOVA with Dunnett’s multiple comparisons test.

Although it enhances neurite growth in some neurons^29,30^, electrical activity represses neurite growth in DRGNs by promoting the activation of Ca_v_1 channels^5,7^. Since caldendrin potentiates the opening of Ca_v_1 channels^14^, diminished Ca_v_1 Ca^2+^ signals in caldendrin KO DRGNs could lessen the inhibitory effects of electrical activity on neurite growth. To test this, we compared the impact of a depolarizing concentration of K^+^ (40 mM, K_40_) on neurite growth in WT and caldendrin KO DRGNs (Fig. 6A). As expected, K_40_ caused a significant reduction (~ 41%) in the number of DRGNs with neurites in cultures from WT males and females, and this was abrogated by co-treatment with the Ca_v_1 blocker isradipine. In contrast, there was no significant effect of K_40_ on DRGNs from caldendrin KO females at either time point (Fig. 6A). Remarkably, these results were not reproduced in cultures from male mice in that K_40_ caused a similar reduction (~ 53%) in the number of DRGNs with neurites in cultures from WT and caldendrin KO males (Fig. 6B). These results indicate that caldendrin couples Ca_v_1 channels to the activity-dependent repression of neurite growth in females but not in males.

**Figure 7:**
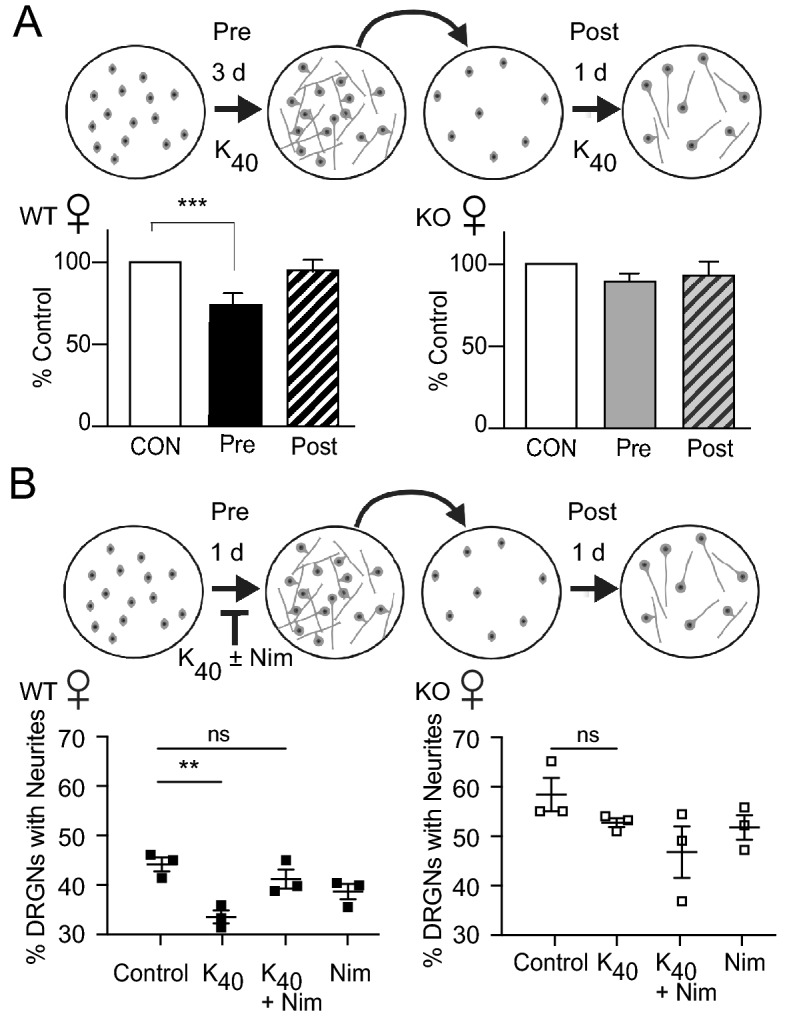
Caldendrin mediates coupling of Ca_v_1 channels to the transcription-dependent phase of neurite growth. (**A**) Top panel, schematic of the experimental design indicating exposure of cultures to K_40_ either before or after replating. Bottom panel, longest neurite per DRGN was measured in control and experimental groups. ***p = 0.0002, F(2,6) = 49.96 by one-way ANOVA, Holm-Sidak’s multiple comparisons test. (**B**) Same as in A except that DRGNs were exposed to K_40_ ± nimodipine (Nim, 10 µM) before (1d) or after replating. % DRGNs with neurites was determined as in Fig. 5. Each data point represents the average determined from ~ 400 DRGNs per culture. **p = 0.004, F(3,8) = 8.3 determined by one-way ANOVA with Dunnett’s multiple comparisons test.

In many neurons, Ca_v_1 channels transduce the effects of electrical activity into changes in gene transcription (i.e., excitation-transcription (E-T) coupling^31^. In DRGNs from female mice, caldendrin may be required for E-T coupling mechanisms that repress neurite growth. If so, then transcription-dependent neurite growth in DRGNs from caldendrin KO mice should be insensitive to depolarization. To test this prediction, we compared the effects of K_40_ before and after replating in the in vitro axotomy assay (Fig. 7A). As expected, K_40_ incubation before but not after replating significantly inhibited neurite regeneration in cultures from WT females. In contrast, K_40_ had no such effect in cultures from caldendrin KO females (Fig. 7A). These results confirm that caldendrin mediates the inhibitory effects of depolarization on neurite growth in DRGNs from female mice. To test whether this effect of caldendrin depended on Ca_v_1 channels, we incubated cultures with the Ca_v_1 blocker nimodipine prior to replating. Nimodipine blunted the inhibitory effect of K_40_ on neurite growth in cultures from WT females but had no effect in cultures from caldendrin KO females (Fig. 7B). Collectively, our findings suggest that caldendrin couples Ca_v_1 channels to activity-dependent, transcriptional pathways that repress neurite regeneration in DRGNs in females but not in males.

## Discussion

During embryonic development, neurons in the central nervous system extend their axons often over long distances to innervate their targets but later lose this capability, presumably because regenerative signaling pathways become silenced^32^. In DRGNs, severing the peripheral neurite can re-activate the genetic program that promotes growth of both the peripherally and centrally directed neurite^25^. Thus, DRGNs represent an excellent model in which to study the mechanisms that normally suppress neurite regeneration. Upon dissociation from adult animals, these neurons readily regrow neurites within hours in culture. Our findings that, compared to in WT cultures, DRGNs in caldendrin KO cultures regenerate their neurites more robustly in standard and explant cultures (Fig. 5) and following in vitro axotomy (Fig. 3) implicate caldendrin in the pathway that opposes regenerative neurite growth.

In the replating assay, the increase in neurite lengths in caldendrin KO vs WT cultures (Fig. 3) suggests that caldendrin suppresses the initial regeneration of neurites (*i.e.,* transcription-dependent phase) and/or the elongation of established neurites (*i.e.,* microtubule-dependent phase)^26^. Like CaM, caldendrin interacts with a variety of effector molecules, such as the Ca_v_1 channel, which could impinge on the transcriptional regulation of neurite growth dynamics^5,7^. Caldendrin and shorter CaBP1 variants bind to the pore-forming subunit of the major Ca_v_1 subtypes expressed in the nervous system, Ca_v_1.2 and Ca_v_1.3, and this interaction potentiates channel opening^14,18,19,33^. Ca_v_1-dependent Ca^2+^ signals are expected to be abnormally low in the absence of caldendrin^21^ and thus may be insufficient to oppose the transcription of regeneration associated genes such as GAP43. In this regard, it is noteworthy that caldendrin levels were low in WT DRGNs at timepoints when GAP43 was high (Fig. 4A). Caldendrin could inhibit the initial regeneration of neurites by enhancing the activity/expression of transcriptional repressors such as RE1 Silencing Transcription Factor (REST), which suppress transcription of genes that promote axon outgrowth^34^. In hippocampal neurons, caldendrin binds to and regulates the nuclear localization of Jacob, a protein that controls cAMP-response element binding protein (CREB)- dependent synaptic remodeling^24^. Interactions between CREB and REST pathways have been reported in neuronal gene regulation networks^35^ but whether these interactions occur in DRGNs has not been investigated. At later stages of neurite growth involving microtubule-dependent neurite elongation, the interaction of caldendrin with LC3, a subunit of microtubule-associated protein (MAP) 1A and 1B^36^, may be important. While the functional significance of this interaction remains to be investigated, caldendrin binding to LC3 could impair phosphorylation of MAP1B which is expected to constrain neurite outgrowth^37^.

In DRGN cultures, the involvement of Ca_v_1 Ca^2+^ signals in neurite growth is readily revealed by depolarizing concentrations of K^+^ which repress neurite growth in a manner that is reversed by Ca_v_1 blockers^5,7^. The absence of any effect of K_40_ on neurite growth in DRGNs from female caldendrin KO mice (Fig. 6A) indicates a critical role for caldendrin in coupling Ca_v_1 channels to activity-dependent neurite growth repression. The fact that this was only applicable to cultures from female and not male caldendrin KO mice was intriguing considering that sex differences have been noted in the transcriptome of mouse DRGNs^38^ and in the responses to peripheral nerve injury in animals and humans^39–41^. This result was not due to major differences in caldendrin protein levels in WT males and females (Fig. 1A). One possibility is that circulating sex steroids in female mice such as estradiol somehow promote caldendrin interactions with Ca_v_1 channels. Estradiol has cell type-specific modulatory effects on Ca_v_1 channels^42–45^ and so could modify channel gating mechanisms that are targeted by caldendrin.

An important follow-up question is how does caldendrin regulate neurite growth in DRGNs from males if Ca_v_1 channels are not involved? Like the shorter CaBP1 variants, caldendrin could bind to and inhibit inositol 1,4,5 trisphosphate receptors (IP_3_Rs)^46,47^ and therefore IP_3_R-mediated Ca^2+^ signals that are known to enhance DRGN neurite outgrowth^48^. Alternatively, caldendrin might interact with the actin-binding protein cortactin^17^ and A-kinase anchoring proteins (AKAPs)^49^, both of which have been implicated in neurite growth regulation^50,51^.

Our findings corroborate and extend our previous work showing that caldendrin represses neurite regeneration in spiral ganglion neurons^21^. In standard cultures obtained from caldendrin KO mice, the initial regrowth of neurites is impaired in DRGNs (Fig. 5B) and spiral ganglion neurons^21^ from caldendrin KO mice. Unlike in our present study of DRGNs (Fig. 5C), the rate of neurite outgrowth in cultures of spiral ganglion neurons was not affected by caldendrin knockdown. This difference could be related to the fact that our previous study utilized spiral ganglion neurons from neonatal (P3-7) mice whereas DRGNs from adult mice were used in the current study. In addition, spiral ganglion neurons generally do not arborize as extensively as DRGNs in culture^52^ and so may have distinct mechanisms controlling outgrowth of established neurites as compared to DRGNs. It is noteworthy that the effect of caldendrin knockdown on high K^+^-induced repression of neurite growth of spiral ganglion neurons from neonatal mice of mixed sexes^21^ was like that we observed for DRGNs from female mice (Fig. 6A). While further studies are necessary, caldendrin could regulate coupling of Ca_v_1 channels to neurite growth repression in sensory neurons of both males and females prior to the age of sexual maturation.

In summary, we have established a novel role for caldendrin in a signaling pathway that represses neurite regeneration in DRGNs in vitro. Establishing the significance of this pathway in peripheral nerve injury models in vivo, as well as an understanding of the mechanisms differentiating this pathway in males and females, remain important challenges for future studies.

## Methods

### Animals

All methods were carried out in accordance with the relevant guidelines and protocols stated in the ARRIVE guidelines^53^. In addition, all protocols were approved by the Institutional Animal Care and Use Committee at the University of Iowa and University of Texas-Austin. Mice (4–8 week old males and females) were housed under a standard 12-h light/dark cycle with access to food and water ad libitum. The caldendrin KO mouse strain (RRID: MGI: 5780462) was described previously^15^ and maintained on a C57BL/6 (Envigo) background. Wild-type (WT) mice were age- and sex- matched C57BL/6 mice. For DRG explant experiments, caldendrin KO/Pirt-GcaMP3 mice were generated by breeding caldendrin KO mice with a mouse strain that expresses the genetically encoded Ca^2+^ indicator GCaMP3 in almost all DRGNs (Pirt-GCaMP3^54^). Prior to dissection of DRGs, mice were anesthetized in a narcosis chamber with isoflurane and subjected to cervical dislocation and decapitation.

### Generation of adenoassociated viruses (AAVs)

The pFBAAVCAGmcsmRubyBgHpA plasmid was generated by cloning a gBlock encoding mRuby (Integrated DNA Technologies) into the G0345 pFBAAVCAGmcsBgHpA plasmid (University of Iowa Viral Vector Core) using Hi-Fi DNA Assembly Master Mix (#E2621, New England Biolabs). pFBAAVCAGmcsCaldendrin-mRubyBgHpA was generated by cloning a gBlock encoding mouse caldendrin (Genbank KJ364651.1) and a 7-amino acid linker into the pFBAAVCAGmcsmRubyBgHpA plasmid. Following diagnostic restriction digest analysis and sequencing, the plasmids were amplified by a commercial source (GENEWIZ). The pFBAAVCAGmcsmRubyBgHpA and pFBAAVCAGmcsCaldendrin-mRubyBgHpA plasmids were used to generate AAV2/PHP.S-CAG-mRuby (AAV-mRuby) and AAV2/PHP.S-CAG-Caldendrin-mRuby (AAV-Caldendrin-mRuby), respectively (~ 1 × 10^12^ viral genomes (vg)/ml). AAV2/PHP.S was used since this serotype strongly transduces DRGNs^55^.

### Western blots

Five lumbar WT and caldendrin KO DRGs were harvested from 4–8-week-old male and female mice, flash frozen in liquid nitrogen, and lysed in lysis buffer (50 mM Tris–HCl pH 7.4, 150 mM NaCl, 0.5% Triton X100, 0.5% n-Dodecyl-beta-Maltoside detergent (Thermofisher Cat# 89,902), Protease Inhibitor Cocktail (Roche cOmplete, EDTA-free (Cat# 05056489001)) and 1 mM PMSF. Plastic pestles were used to homogenize the pellet in a 1.5 ml microfuge tube. Lysates were cleared by spinning down samples at 14,000 × g at 4 °C for 10 min. NuPAGE™ LDS Sample Buffer (4X) (Cat# NP0007, ThermoFisher) was added to lysates which were then incubated at 65 °C for 10 min. The lysate (25% of total) was loaded into a 4–20% Tris–Glycine gel (Invitrogen Cat# XP04200BOX), run in Tris–Glycine SDS Running buffer (Novex Life technologies Cat# LC2675) and transferred overnight using Tris–Glycine Transfer Buffer (Novex Life technologies Cat# LC3675). Blots were incubated in blocking buffer (5% fat free milk in TBST) for 30 min prior to incubation for 1 h in primary antibodies diluted in blocking buffer: Goat anti-mouse GAPDH (RRID: AB_1078991, Sigma-Aldrich) 1:1000, goat anti-mouse GAP43 (RRID: AB_2533123, Thermo Fisher Scientific) 1:1000, rabbit polyclonal antibody against caldendrin and goat anti-mouse β-tubulin (RRID: AB_396360, BD Biosciences) 1:3000. Chemiluminescent detection was accomplished with a Li-COR Odyssey Imager by exposing the blot for 10 min. Image Studio lite software was used to prepare images.

### Preparation of dissociated DRGNs and DRG explants

DRGN cultures were prepared as previously^56^. One day before culture preparation, 14 mm round glass cover slips (Deckglaser) were placed in 24 well plates (Cat# 229123, Celltreat) and coated with poly-L-ornithine (0.1 mg/ml in 10 mM borate buffer, pH 8.4, P4957, Sigma) overnight at 37 °C. Coverslips were rinsed with Milli-Q H_2_O three times and coated with laminin (20 μg/ml in ultra-pure H_2_O, 11243217001, Sigma) for 1 h at 37 °C. DRGs (20–30) were harvested in Neurobasal™ media (Cat#21103049, ThermoFisher) and DRGNs were dissociated in 0.125% Trypsin–EDTA (Cat #25200056, ThermoFisher) and 0.1% collagenase type I (1 mg/mL in HBSS without calcium or magnesium (−/−)), (Cat #17100, ThermoFisher) for 40 min. After the enzyme incubation, 10% horse serum was added to inactivate the proteases, the cell suspension was centrifuged at 500 rcf for 5 min. The neurons were resuspended in Neurobasal™ media supplemented with 2.5% L-glutamine (Cat #25030081, ThermoFisher) and 2% N-21 (Cat #SCM081, Millipore Sigma), plated at a density of 5 ganglia per well, and cultured for 18, 24 and 48 h in a humidified incubator at 37 °C with 5% CO_2_. In the replating assay^26^, DRGNs were cultured for 72 h prior to dissociation with TrypLE™ Express (Cat#12604013, ThermoFisher) for 1 min and replating and culture for an additional 24 h. Dichlorobenzimidazole riboside (DBR, 40 µM; Cat# D1916 Sigma-Aldrich) or nocodazole (50 µM, Cat# M1404 Sigma-Aldrich) was diluted in dimethylsulfoxide (DMSO). Drug-containing solution or control solution (0.1% DMSO) was added to the culture medium either before or after replating. In some experiments, DRGNs were depolarized with 40 mM KCl in the presence or absence of Ca_v_1 channel blockers (isradipine, Cat#2004, Tocris, or nimodipine (each at 10 μM), Cat#0600, Tocris). For the rescue experiments in Fig. 4C,D, AAV (1 μl, 1 × 10^12^ vg/ml) was added to each well of a 24 well plate 4 h after plating the DRGNs. After 72 h, the DRGNs were replated and cultured for an additional 24 h and processed for immunofluorescence.

For DRG explant cultures, lumbar DRGs were dissected from male PirtGCaMP3 and caldendrin KO/ PirtGCaMP3 mice (4–5 weeks old). To facilitate directional growth of neurites, DRG explants were cultured on surfaces of 40 wt% hexyl methacrylate (SigmaAldrich) and 60 wt% 1,6–hexanediol dimethacylate (SigmaAldrich) slides in which micropatterned grooves (8–9 μm amplitude, periodicity of 50 μm) were generated by photopolymerization^57,58^. The surfaces were coated with poly-L-ornithine as described for DRGN cultures and explants. Each explant was placed into a cloning cylinder (Bellco 2090-01010) on the slide and maintained in DMEM (Cat #A1896701, ThermoFisher) media supplemented with 2.5% L-glutamine (Cat #25030081, ThermoFisher) and 2% N-21 (Cat #SCM081, Millipore Sigma) in a humidified incubator at 37 °C with 5% CO_2_. DRG explants were imaged at 24, 48, and 72 h using a Leica DMRIII epifluorescence microscope and a cooled CCD camera with Leica FW4000 software. The culture media was changed daily.

### Immunofluorescence

For analysis of caldendrin expression in DRGs, lumbar DRGs (L4-L6) were harvested from male and female adult WT mice and immersed in a 4% paraformaldehyde (PFA) in phosphate-buffered saline (PBS, Cat #AAJ19943K2, ThermoFisher) and stored at 4 °C overnight prior to transfer to 30% sucrose solution in PBS for an additional 24 h. The fixed tissue was molded in Optimal Cutting Temperature compound (Sakura Finetek) and 15-μm thick sections were obtained using a cryostat (Leica). Sections were mounted on charged slides and dried on a slide warmer for 5 min. Sections were then subject to rinsing, blocking, and antibody incubations at room temperature (RT). After rinsing in PBS and incubating in blocking buffer (10% normal goat serum, Cat #PCN5000 and 0.2% Triton™ X-100 Cat #9002-93-1, ThermoFisher, in PBS) for 30 min, the sections were incubated for 1 h in the following primary antibodies diluted 1:1000 in blocking buffer: chicken polyclonal antibody anti-neurofilament 200 (NF200, RRID: AB_2313552, Aves); chicken polyclonal antibody anti-Peripherin (RRID:AB_10785694, Aves Labs); rabbit polyclonal antibody against caldendrin (UW72)^59^. Sections were washed 3 times with PBS for 10 min followed by incubation with secondary antibodies diluted 1:500 in blocking buffer: goat anti-Chicken Alexa Fluor 546 (RRID: AB_2534097, ThermoFisher) and goat anti-Rabbit Alexa Fluor 488 (RRID:AB_2534096, ThermoFisher). Sections were then washed 3 times with PBS for 10 min each and mounted using Fluoromount-G (SouthernBiotech) and allowed to cure for 24 h in the dark before imaging. DRGs from at least 4 mice were processed in 4 independent experiments. At least 3 sections were analyzed using an Olympus Fluoview 1000 confocal laser scanning microscope with a 20 × objective and FluoView software (RRID: SCR_014215). For quantitation of the percentage of DRGNs exhibiting colocalized caldendrin and NF200 or peripherin fluorescence (reported in the text), the number of double-labeled cells was evaluated in confocal images by researchers blinded to the labeling conditions and genotype.

Immunofluorescence of dissociated DRGNs was done 18–48 h after plating or 24 h post-replating in the in vitro axotomy experiments. The culture medium was removed and the coverslips washed with PBS. All subsequent steps were done at room temperature. DRGNs were fixed by adding 4% PFA for 10 min and washed with PBS (3 times, 5 min each). The coverslips were then incubated in blocking buffer for 30 min, and subsequently for 1 h in chicken polyclonal antibody against NF200 (RRID: AB_2313552, Aves) diluted 1:1000 in 5% normal goat serum (Cat #PCN5000, ThermoFisher), 0.5% TritonTM X-100 (Cat #9002–93-1, Fisher Scientific). Each coverslip was washed 3 times for 5 min with PBS followed by addition of Goat anti-Rabbit Alexa Fluor®488 (RRID:AB_2534096, ThermoFisher) (1:1000 in blocking buffer) for 1 h in the dark. To stain nuclei, the coverslips were incubated with Hoechst (DAPI1:10,000 in PBS, Cat # 62,249, ThermoFisher) for 5 min. The DRGNs were washed with PBS (3 times, 5 min each). The coverslips were mounted using Fluoromount-G® (SouthernBiotech) and cured for 24 h in the dark. Images were obtained using an Olympus BX53 microscope with a 20 × objective equipped with Olympus DP72 camera and CellSens Standard imaging software (RRID: SCR_014551).

### Quantitative analysis of neurite growth

All quantitative analysis was performed on immunofluorescence images by researchers blinded to experimental conditions. In DRGN cultures, only the NF200-positive neurons with Hoechst-positive nuclei were included in the analyses. The % of DRGNs with neurites was determined as: (# of DRGNs with a neurite at least as long as the diameter of the cell soma) / (# of DRGNs on the coverslip) × 100. For each experiment, 300–400 neurons per coverslip were analyzed in 1 coverslip per animal (at least 3 WT or caldendrin KO mice per experiment). In some analyses, the longest axon was measured using Fiji (ImageJ) plug-in NeuronJ^60^. In these experiments, at least 15–30 DRGN images were analyzed.

For analyses in DRG explants, an in-house developed custom tool facilitated semi-automated analysis of the entire length of the individual neurites and subsequent calculation of the composite neurite length for each image of a DRG explant. Dijkstra's algorithm^61^ was applied to find a path with the minimum accumulated edge cost between two interactively identified image locations, each positioned on the neurite. These interactively defined nodes served as neurite component start- and end-locations in a 2-dimensional graph consisting of graph nodes corresponding to all image pixels and inter-pixel 8-neighbor edges. The mean brightness value difference of neighboring pixel-pairs defined graph edge costs throughout the graph. To avoid the tracing error caused by complex neurite structures, multiple segments of the individual neurite were determined sequentially. For each image of a DRG explant, the semi-automated analysis enabled tracing of the entire length of the individual neurites and subsequent calculation of neurite length. The maximal neurite lengths were measured in at least two DRG explants per animal (n = 3 animals) and the averages were plotted against time in culture. The rate of neurite growth was determined by a linear fit of these data.

### Experimental design and statistical analysis

Graphs were generated and statistical analysis was done with Graphpad Prism 9 software (RRID: SCR_002798, GraphPad Software, San Diego, CA). Data were first tested for normality by Shapiro- Wilk normality test. If normally distributed, the data were analyzed by unpaired t-test or ANOVA with Sidak multiple comparison test with a single pool variance. If not normally distributed, the data were analyzed by Mann–Whitney test. For two-way ANOVA tests with main effects only, Tukey’s multiple comparisons test was performed with individual variances computed for each comparison. Otherwise, post hoc tests were performed, and simple main effects were reported using adjusted p value for multiple comparisons. Data are presented as mean and SEM and *p* ≤ 0.05 was considered statistically significant.

## Supplementary Information

Supplementary Information.

## Data Availability

The datasets generated during and/or analyzed during the current study are available from the corresponding author on reasonable request.
