# Supplementary material for: Caldendrin represses neurite regeneration and growth in dorsal root ganglion neurons
Source: Sci Rep. 2023 Feb 14;13:2608. doi: 10.1038/s41598-023-29622-9 (PMC9929226; doi:10.1038/s41598-023-29622-9)
Supplement: Supplementary file 1 — Supplementary Information. [file 41598_2023_29622_MOESM1_ESM.pdf]

Supplementary Figure 1

A

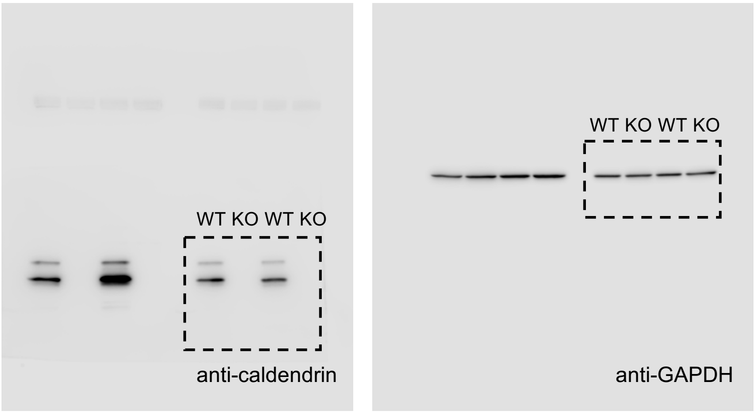

B

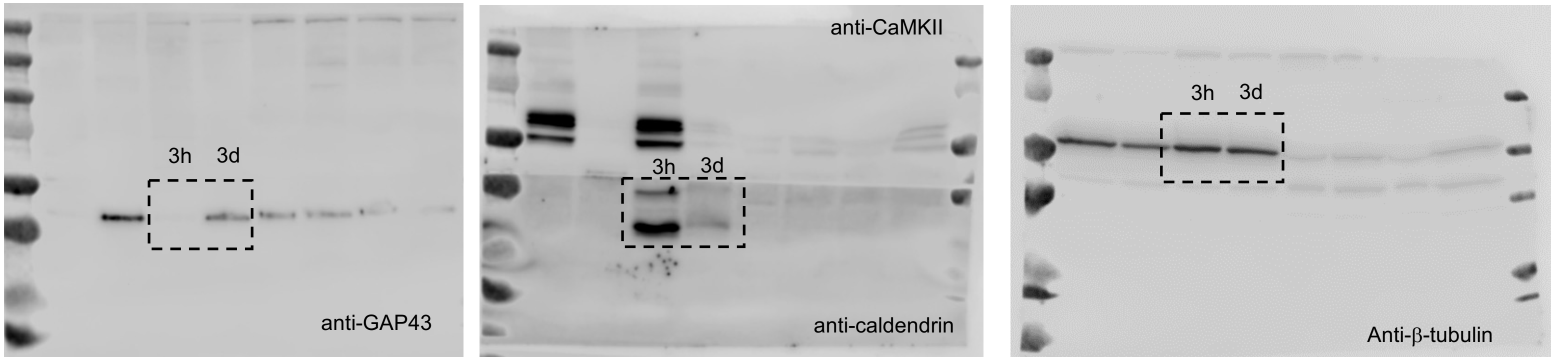

C

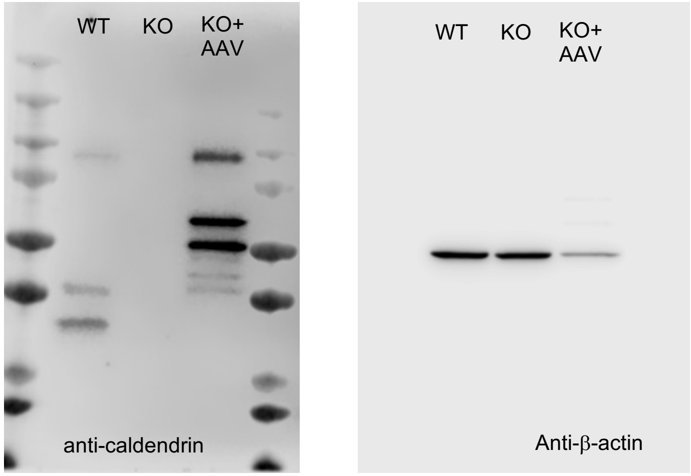

Supplementary Figure 1. *A-C*, Full-length blots for images of western blots shown in Fig.1A (A), Fig.4A (B), and Fig.4B (C) of the main text. Boxed regions correspond to cropped regions shown in the figures. Antibodies used for blots are indicated. In *B*, top portion of the blot was probed with anti-CaMKII antibodies (results were not incorporated into the text).

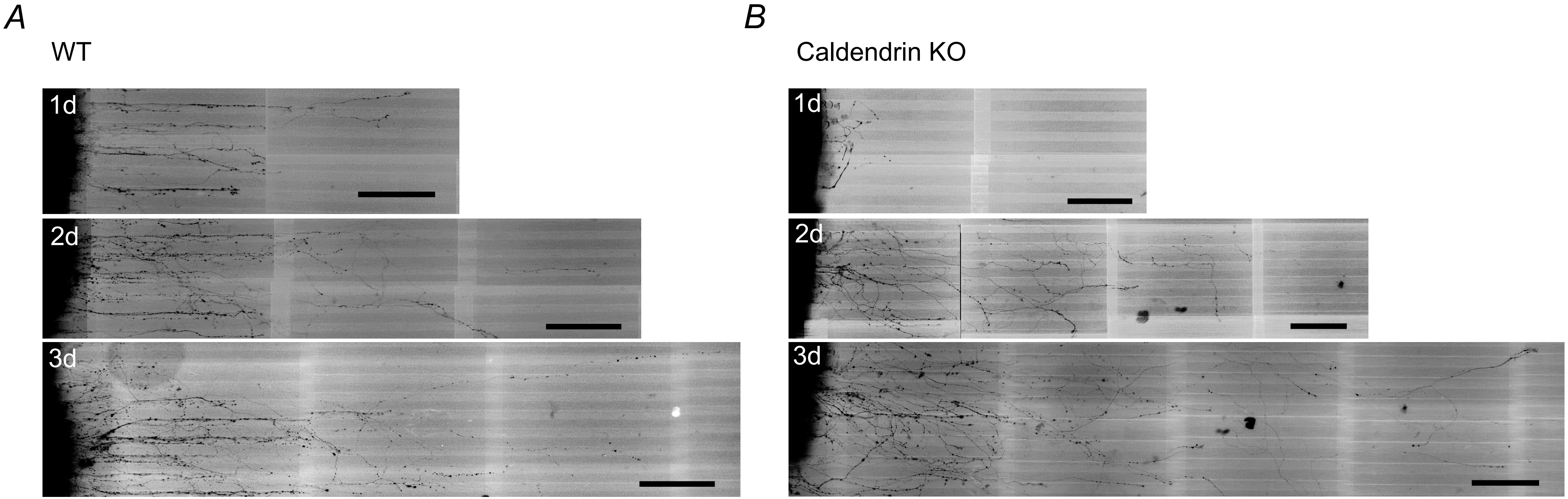

Supplementary Figure 2. Representative images of DRG explant cultures used for data analyzed in Fig.5C of the main text. Lumbar DRGs from WT Pirt-GcaMP3 (A) or Caldendrin KO Pirt-GcaMP3 (B) mice were plated on micropatterned substrates and subject to live imaging every 24 hours for 3 days during which neurite lengths were measured. Panels represent the same explant imaged over the 3 days. Scale bar, 161 µm.
